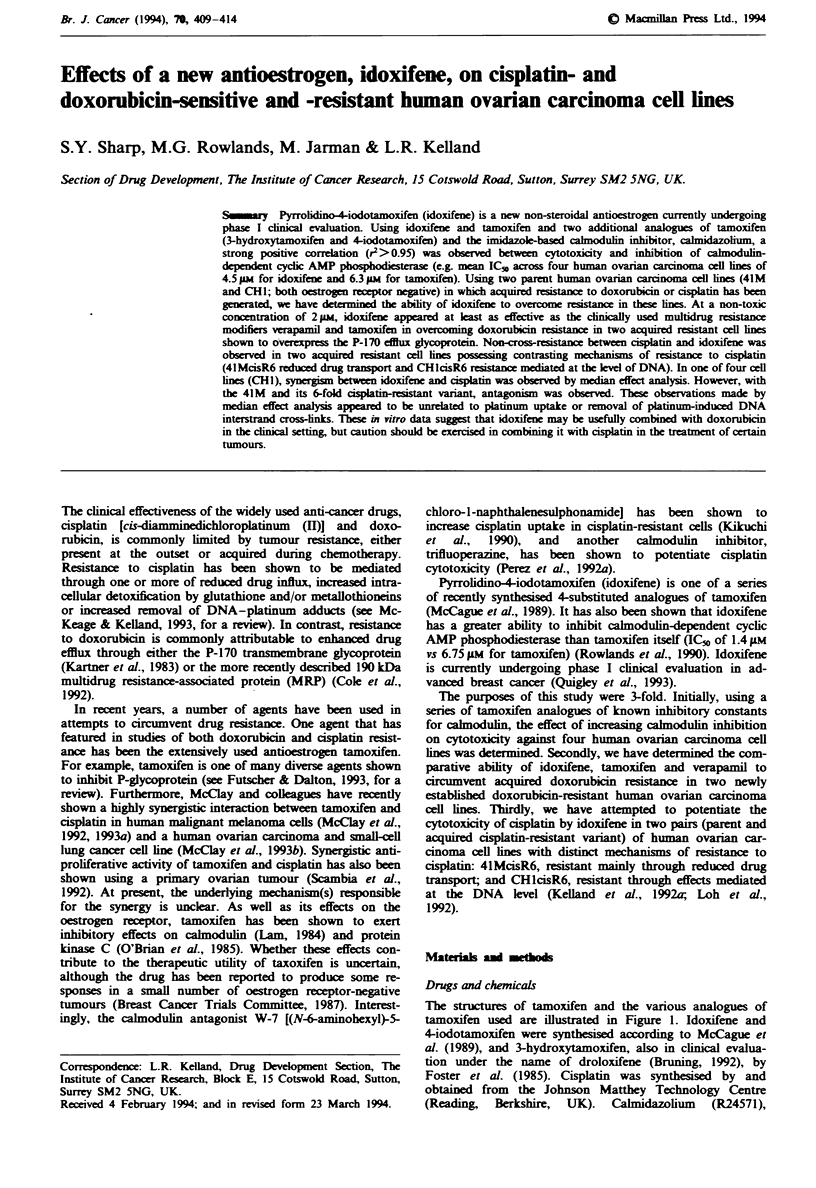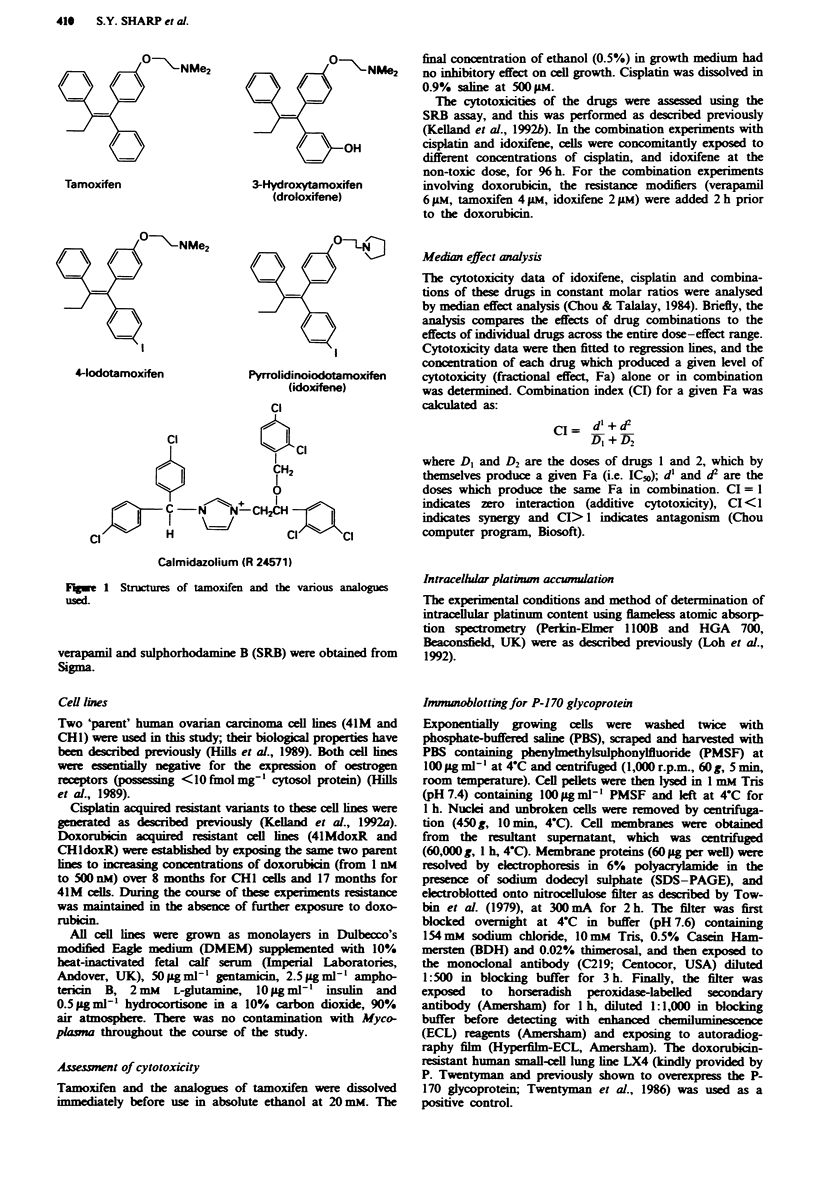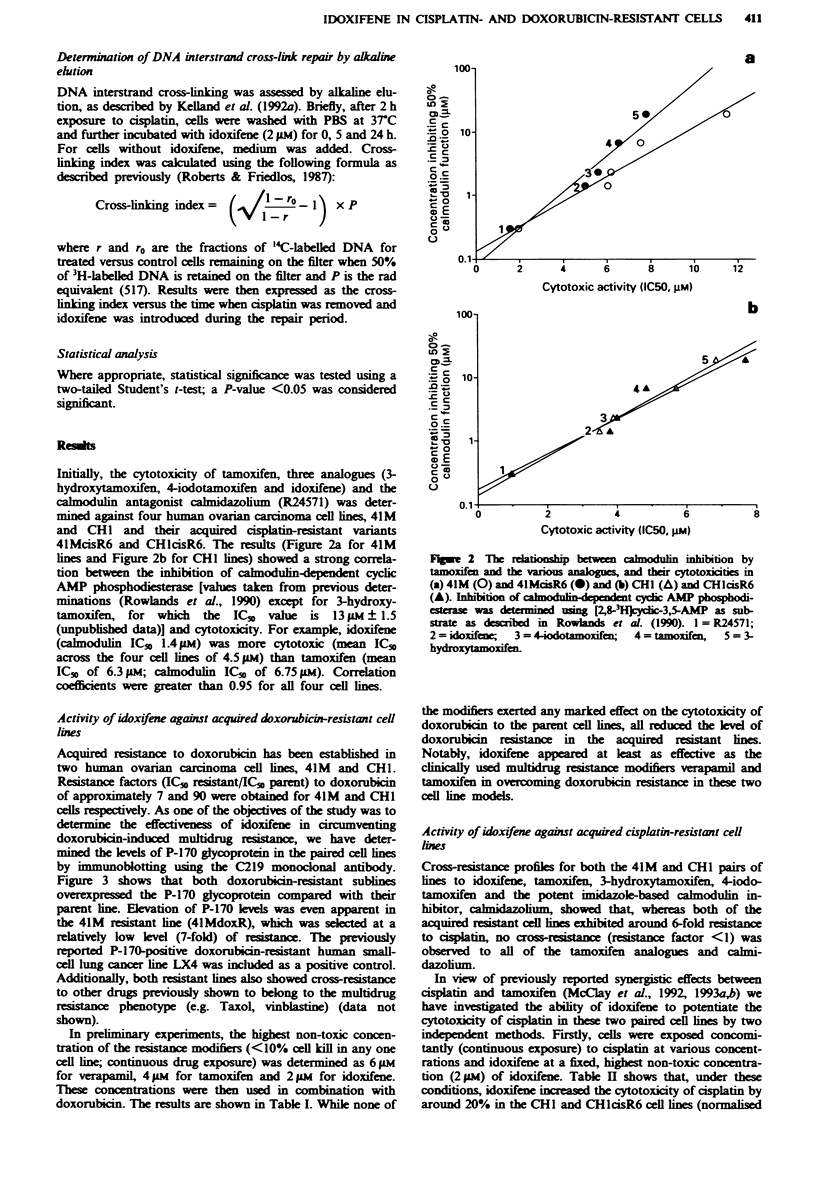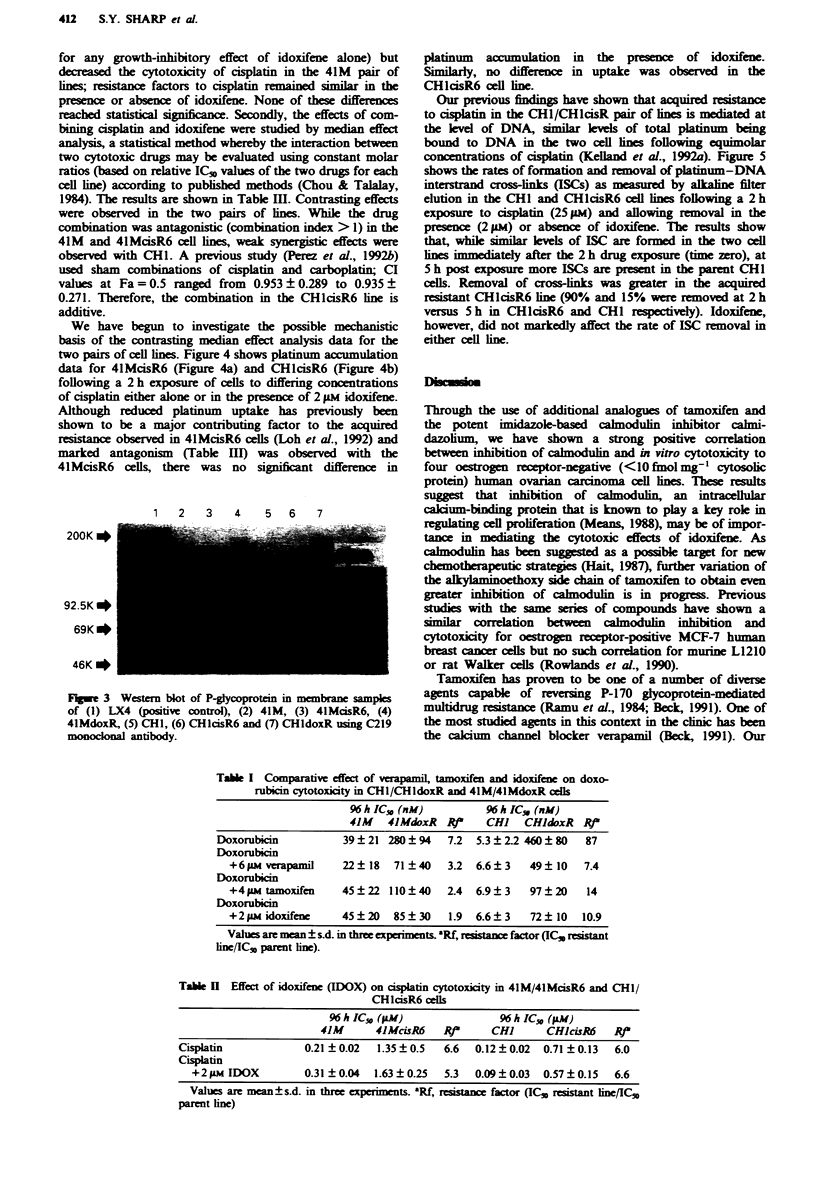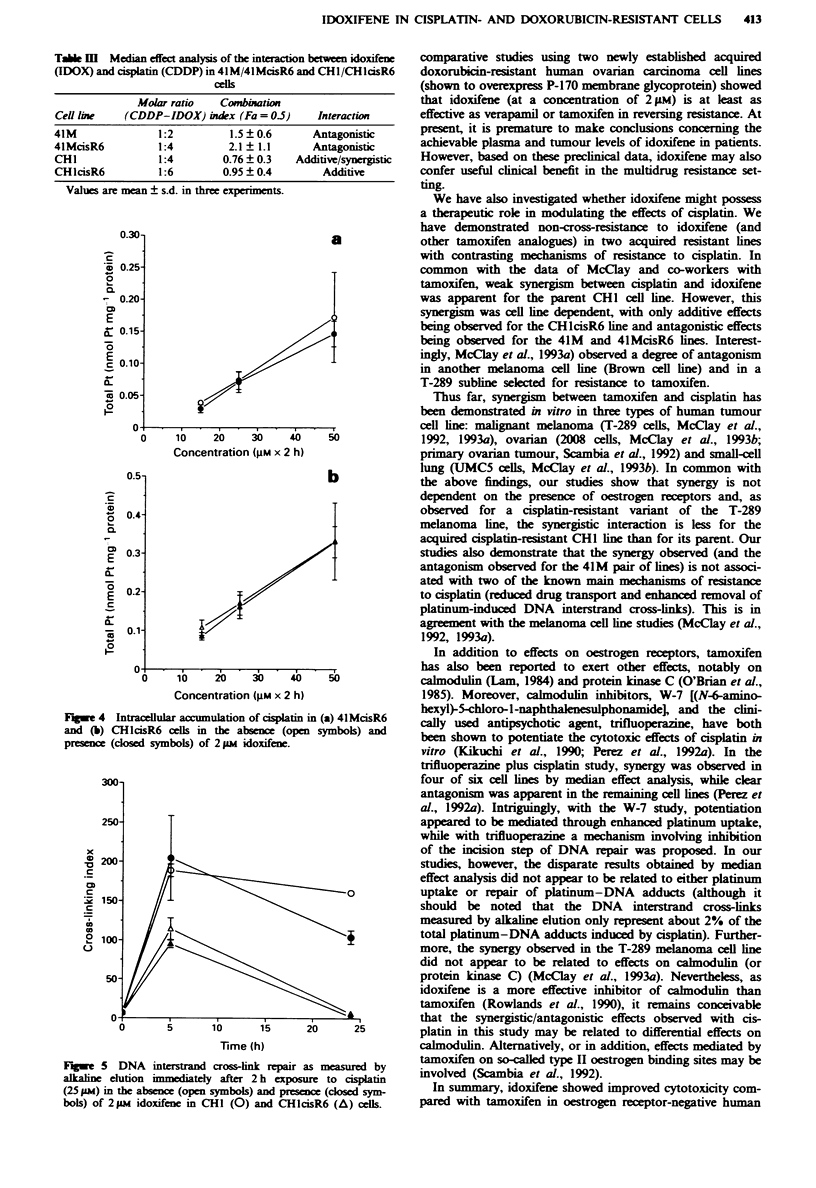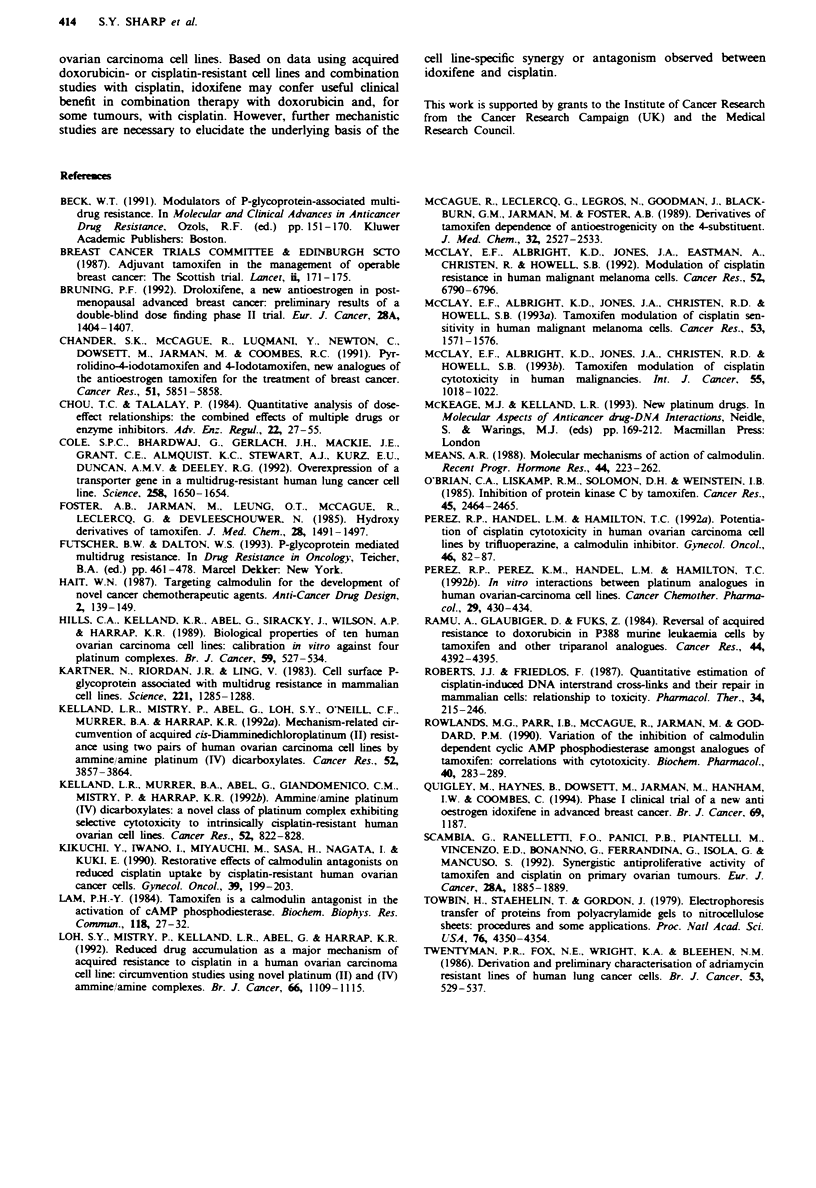# Effects of a new antioestrogen, idoxifene, on cisplatin- and doxorubicin-sensitive and -resistant human ovarian carcinoma cell lines.

**DOI:** 10.1038/bjc.1994.319

**Published:** 1994-09

**Authors:** S. Y. Sharp, M. G. Rowlands, M. Jarman, L. R. Kelland

**Affiliations:** Section of Drug Development, Institute of Cancer Research, Sutton, Surrey, UK.

## Abstract

**Images:**


Br. J. Cancer (1994), 70, 409-414 © Macmillan Press Ltd., 1994

### Effects of a new antioestrogen, idoxifene, on cisplatin- and doxorubicin-sensitive and -resistant human ovarian carcinoma cell lines

S.Y. Sharp, M.G. Rowlands, M. Jarman & L.R. Kelland

*Section of Drug Development, The Institute of Cancer Research, 15 Cotswold Road, Sutton, Surrey SM2 5NG, UK.*

**Summary** Pyrrolidino-4-iodotamoxifen (idoxifene) is a new non-steroidal antioestrogen currently undergoing phase I clinical evaluation. Using idoxifene and tamoxifen and two additional analogues of tamoxifen (3-hydroxytamoxifen and 4-iodotamoxifen) and the imidazole-based calmodulin inhibitor, calmidazolium, a strong positive correlation ($r^2 > 0.95$) was observed between cytotoxicity and inhibition of calmodulin-dependent cyclic AMP phosphodiesterase (e.g. mean $IC_{50}$ across four human ovarian carcinoma cell lines of 4.5 μM for idoxifene and 6.3 μM for tamoxifen). Using two parent human ovarian carcinoma cell lines (41M and CH1; both oestrogen receptor negative) in which acquired resistance to doxorubicin or cisplatin has been generated, we have determined the ability of idoxifene to overcome resistance in these lines. At a non-toxic concentration of 2 μM, idoxifene appeared at least as effective as the clinically used multidrug resistance modifiers verapamil and tamoxifen in overcoming doxorubicin resistance in two acquired resistant cell lines shown to overexpress the P-170 efflux glycoprotein. Non-cross-resistance between cisplatin and idoxifene was observed in two acquired resistant cell lines possessing contrasting mechanisms of resistance to cisplatin (41McisR6 reduced drug transport and CH1cisR6 resistance mediated at the level of DNA). In one of four cell lines (CH1), synergism between idoxifene and cisplatin was observed by median effect analysis. However, with the 41M and its 6-fold cisplatin-resistant variant, antagonism was observed. These observations made by median effect analysis appeared to be unrelated to platinum uptake or removal of platinum-induced DNA interstrand cross-links. These *in vitro* data suggest that idoxifene may be usefully combined with doxorubicin in the clinical setting, but caution should be exercised in combining it with cisplatin in the treatment of certain tumours.

The clinical effectiveness of the widely used anti-cancer drugs, cisplatin [*cis*-diamminedichloroplatinum (II)] and doxorubicin, is commonly limited by tumour resistance, either present at the outset or acquired during chemotherapy. Resistance to cisplatin has been shown to be mediated through one or more of reduced drug influx, increased intracellular detoxification by glutathione and/or metallothioneins or increased removal of DNA-platinum adducts (see McKeage & Kelland, 1993, for a review). In contrast, resistance to doxorubicin is commonly attributable to enhanced drug efflux through either the P-170 transmembrane glycoprotein (Kartner *et al.*, 1983) or the more recently described 190 kDa multidrug resistance-associated protein (MRP) (Cole *et al.*, 1992).

In recent years, a number of agents have been used in attempts to circumvent drug resistance. One agent that has featured in studies of both doxorubicin and cisplatin resistance has been the extensively used antioestrogen tamoxifen. For example, tamoxifen is one of many diverse agents shown to inhibit P-glycoprotein (see Futscher & Dalton, 1993, for a review). Furthermore, McClay and colleagues have recently shown a highly synergistic interaction between tamoxifen and cisplatin in human malignant melanoma cells (McClay *et al.*, 1992, 1993*a*) and a human ovarian carcinoma and small-cell lung cancer cell line (McClay *et al.*, 1993*b*). Synergistic antiproliferative activity of tamoxifen and cisplatin has also been shown using a primary ovarian tumour (Scambia *et al.*, 1992). At present, the underlying mechanism(s) responsible for the synergy is unclear. As well as its effects on the oestrogen receptor, tamoxifen has been shown to exert inhibitory effects on calmodulin (Lam, 1984) and protein kinase C (O'Brian *et al.*, 1985). Whether these effects contribute to the therapeutic utility of taxoxifen is uncertain, although the drug has been reported to produce some responses in a small number of oestrogen receptor-negative tumours (Breast Cancer Trials Committee, 1987). Interestingly, the calmodulin antagonist W-7 [(*N*-6-aminohexyl)-5-chloro-1-naphthalenesulphonamide] has been shown to increase cisplatin uptake in cisplatin-resistant cells (Kikuchi *et al.*, 1990), and another calmodulin inhibitor, trifluoperazine, has been shown to potentiate cisplatin cytotoxicity (Perez *et al.*, 1992*a*).

Pyrrolidino-4-iodotamoxifen (idoxifene) is one of a series of recently synthesised 4-substituted analogues of tamoxifen (McCague *et al.*, 1989). It has also been shown that idoxifene has a greater ability to inhibit calmodulin-dependent cyclic AMP phosphodiesterase than tamoxifen itself ($IC_{50}$ of 1.4 μM *vs* 6.75 μM for tamoxifen) (Rowlands *et al.*, 1990). Idoxifene is currently undergoing phase I clinical evaluation in advanced breast cancer (Quigley *et al.*, 1993).

The purposes of this study were 3-fold. Initially, using a series of tamoxifen analogues of known inhibitory constants for calmodulin, the effect of increasing calmodulin inhibition on cytotoxicity against four human ovarian carcinoma cell lines was determined. Secondly, we have determined the comparative ability of idoxifene, tamoxifen and verapamil to circumvent acquired doxorubicin resistance in two newly established doxorubicin-resistant human ovarian carcinoma cell lines. Thirdly, we have attempted to potentiate the cytotoxicity of cisplatin by idoxifene in two pairs (parent and acquired cisplatin-resistant variant) of human ovarian carcinoma cell lines with distinct mechanisms of resistance to cisplatin: 41McisR6, resistant mainly through reduced drug transport; and CH1cisR6, resistant through effects mediated at the DNA level (Kelland *et al.*, 1992*a*; Loh *et al.*, 1992).

### Materials and methods

### *Drugs and chemicals*

The structures of tamoxifen and the various analogues of tamoxifen used are illustrated in Figure 1. Idoxifene and 4-iodotamoxifen were synthesised according to McCague *et al.* (1989), and 3-hydroxytamoxifen, also in clinical evaluation under the name of droloxifene (Bruning, 1992), by Foster *et al.* (1985). Cisplatin was synthesised by and obtained from the Johnson Matthey Technology Centre (Reading, Berkshire, UK). Calmidazolium (R24571),

Correspondence: L.R. Kelland, Drug Development Section, The Institute of Cancer Research, Block E, 15 Cotswold Road, Sutton, Surrey SM2 5NG, UK.
Received 4 February 1994; and in revised form 23 March 1994.

**Figure 1** Structures of tamoxifen and the various analogues used.

verapamil and sulphorhodamine B (SRB) were obtained from Sigma.

*Cell lines*

Two 'parent' human ovarian carcinoma cell lines (41M and CH1) were used in this study; their biological properties have been described previously (Hills *et al.*, 1989). Both cell lines were essentially negative for the expression of oestrogen receptors (possessing <10 fmol mg$^{-1}$ cytosol protein) (Hills *et al.*, 1989).

Cisplatin acquired resistant variants to these cell lines were generated as described previously (Kelland *et al.*, 1992*a*). Doxorubicin acquired resistant cell lines (41MdoxR and CH1doxR) were established by exposing the same two parent lines to increasing concentrations of doxorubicin (from 1 nM to 500 nM) over 8 months for CH1 cells and 17 months for 41M cells. During the course of these experiments resistance was maintained in the absence of further exposure to doxorubicin.

All cell lines were grown as monolayers in Dulbecco's modified Eagle medium (DMEM) supplemented with 10% heat-inactivated fetal calf serum (Imperial Laboratories, Andover, UK), 50 μg ml$^{-1}$ gentamicin, 2.5 μg ml$^{-1}$ amphotericin B, 2 mM L-glutamine, 10 μg ml$^{-1}$ insulin and 0.5 μg ml$^{-1}$ hydrocortisone in a 10% carbon dioxide, 90% air atmosphere. There was no contamination with *Mycoplasma* throughout the course of the study.

*Assessment of cytotoxicity*

Tamoxifen and the analogues of tamoxifen were dissolved immediately before use in absolute ethanol at 20 mM. The final concentration of ethanol (0.5%) in growth medium had no inhibitory effect on cell growth. Cisplatin was dissolved in 0.9% saline at 500 μM.

The cytotoxicities of the drugs were assessed using the SRB assay, and this was performed as described previously (Kelland *et al.*, 1992*b*). In the combination experiments with cisplatin and idoxifene, cells were concomitantly exposed to different concentrations of cisplatin, and idoxifene at the non-toxic dose, for 96 h. For the combination experiments involving doxorubicin, the resistance modifiers (verapamil 6 μM, tamoxifen 4 μM, idoxifene 2 μM) were added 2 h prior to the doxorubicin.

*Median effect analysis*

The cytotoxicity data of idoxifene, cisplatin and combinations of these drugs in constant molar ratios were analysed by median effect analysis (Chou & Talalay, 1984). Briefly, the analysis compares the effects of drug combinations to the effects of individual drugs across the entire dose–effect range. Cytotoxicity data were then fitted to regression lines, and the concentration of each drug which produced a given level of cytotoxicity (fractional effect, Fa) alone or in combination was determined. Combination index (CI) for a given Fa was calculated as:

$$CI = \frac{d^1 + d^2}{D_1 + D_2}$$

where $D_1$ and $D_2$ are the doses of drugs 1 and 2, which by themselves produce a given Fa (i.e. $IC_{50}$); $d^1$ and $d^2$ are the doses which produce the same Fa in combination. CI = 1 indicates zero interaction (additive cytotoxicity), CI<1 indicates synergy and CI>1 indicates antagonism (Chou computer program, Biosoft).

*Intracellular platinum accumulation*

The experimental conditions and method of determination of intracellular platinum content using flameless atomic absorption spectrometry (Perkin-Elmer 1100B and HGA 700, Beaconsfield, UK) were as described previously (Loh *et al.*, 1992).

*Immunoblotting for P-170 glycoprotein*

Exponentially growing cells were washed twice with phosphate-buffered saline (PBS), scraped and harvested with PBS containing phenylmethylsulphonylfluoride (PMSF) at 100 μg ml$^{-1}$ at 4°C and centrifuged (1,000 r.p.m., 60 *g*, 5 min, room temperature). Cell pellets were then lysed in 1 mM Tris (pH 7.4) containing 100 μg ml$^{-1}$ PMSF and left at 4°C for 1 h. Nuclei and unbroken cells were removed by centrifugation (450 *g*, 10 min, 4°C). Cell membranes were obtained from the resultant supernatant, which was centrifuged (60,000 *g*, 1 h, 4°C). Membrane proteins (60 μg per well) were resolved by electrophoresis in 6% polyacrylamide in the presence of sodium dodecyl sulphate (SDS–PAGE), and electroblotted onto nitrocellulose filter as described by Towbin *et al.* (1979), at 300 mA for 2 h. The filter was first blocked overnight at 4°C in buffer (pH 7.6) containing 154 mM sodium chloride, 10 mM Tris, 0.5% Casein Hammersten (BDH) and 0.02% thimerosal, and then exposed to the monoclonal antibody (C219; Centocor, USA) diluted 1:500 in blocking buffer for 3 h. Finally, the filter was exposed to horseradish peroxidase-labelled secondary antibody (Amersham) for 1 h, diluted 1:1,000 in blocking buffer before detecting with enhanced chemiluminescence (ECL) reagents (Amersham) and exposing to autoradiography film (Hyperfilm-ECL, Amersham). The doxorubicin-resistant human small-cell lung line LX4 (kindly provided by P. Twentyman and previously shown to overexpress the P-170 glycoprotein; Twentyman *et al.*, 1986) was used as a positive control.

*Determination of DNA interstrand cross-link repair by alkaline elution*

DNA interstrand cross-linking was assessed by alkaline elution, as described by Kelland *et al.* (1992*a*). Briefly, after 2 h exposure to cisplatin, cells were washed with PBS at 37°C and further incubated with idoxifene (2 μM) for 0, 5 and 24 h. For cells without idoxifene, medium was added. Cross-linking index was calculated using the following formula as described previously (Roberts & Friedlos, 1987):

$$\text{Cross-linking index} = \left(\sqrt{\frac{1-r_0}{1-r}} - 1\right) \times P$$

where $r$ and $r_0$ are the fractions of $^{14}C$-labelled DNA for treated versus control cells remaining on the filter when 50% of $^{3}H$-labelled DNA is retained on the filter and $P$ is the rad equivalent (517). Results were then expressed as the cross-linking index versus the time when cisplatin was removed and idoxifene was introduced during the repair period.

*Statistical analysis*

Where appropriate, statistical significance was tested using a two-tailed Student's *t*-test; a *P*-value <0.05 was considered significant.

### Results

Initially, the cytotoxicity of tamoxifen, three analogues (3-hydroxytamoxifen, 4-iodotamoxifen and idoxifene) and the calmodulin antagonist calmidazolium (R24571) was determined against four human ovarian carcinoma cell lines, 41M and CH1 and their acquired cisplatin-resistant variants 41McisR6 and CH1cisR6. The results (Figure 2a for 41M lines and Figure 2b for CH1 lines) showed a strong correlation between the inhibition of calmodulin-dependent cyclic AMP phosphodiesterase [values taken from previous determinations (Rowlands *et al.*, 1990) except for 3-hydroxytamoxifen, for which the $IC_{50}$ value is 13 μM ± 1.5 (unpublished data)] and cytotoxicity. For example, idoxifene (calmodulin $IC_{50}$ 1.4 μM) was more cytotoxic (mean $IC_{50}$ across the four cell lines of 4.5 μM) than tamoxifen (mean $IC_{50}$ of 6.3 μM; calmodulin $IC_{50}$ of 6.75 μM). Correlation coefficients were greater than 0.95 for all four cell lines.

**Figure 2** The relationship between calmodulin inhibition by tamoxifen and the various analogues, and their cytotoxicities in (**a**) 41M (○) and 41McisR6 (●) and (**b**) CH1 (△) and CH1cisR6 (▲). Inhibition of calmodulin-dependent cyclic AMP phosphodiesterase was determined using [2,8-$^{3}$H]cyclic-3,5-AMP as substrate as described in Rowlands *et al.* (1990). 1 = R24571; 2 = idoxifene; 3 = 4-iodotamoxifen; 4 = tamoxifen, 5 = 3-hydroxytamoxifen.

*Activity of idoxifene against acquired doxorubicin-resistant cell lines*

Acquired resistance to doxorubicin has been established in two human ovarian carcinoma cell lines, 41M and CH1. Resistance factors ($IC_{50}$ resistant/$IC_{50}$ parent) to doxorubicin of approximately 7 and 90 were obtained for 41M and CH1 cells respectively. As one of the objectives of the study was to determine the effectiveness of idoxifene in circumventing doxorubicin-induced multidrug resistance, we have determined the levels of P-170 glycoprotein in the paired cell lines by immunoblotting using the C219 monoclonal antibody. Figure 3 shows that both doxorubicin-resistant sublines overexpressed the P-170 glycoprotein compared with their parent line. Elevation of P-170 levels was even apparent in the 41M resistant line (41MdoxR), which was selected at a relatively low level (7-fold) of resistance. The previously reported P-170-positive doxorubicin-resistant human small-cell lung cancer line LX4 was included as a positive control. Additionally, both resistant lines also showed cross-resistance to other drugs previously shown to belong to the multidrug resistance phenotype (e.g. Taxol, vinblastine) (data not shown).

In preliminary experiments, the highest non-toxic concentration of the resistance modifiers (<10% cell kill in any one cell line; continuous drug exposure) was determined as 6 μM for verapamil, 4 μM for tamoxifen and 2 μM for idoxifene. These concentrations were then used in combination with doxorubicin. The results are shown in Table I. While none of the modifiers exerted any marked effect on the cytotoxicity of doxorubicin to the parent cell lines, all reduced the level of doxorubicin resistance in the acquired resistant lines. Notably, idoxifene appeared at least as effective as the clinically used multidrug resistance modifiers verapamil and tamoxifen in overcoming doxorubicin resistance in these two cell line models.

*Activity of idoxifene against acquired cisplatin-resistant cell lines*

Cross-resistance profiles for both the 41M and CH1 pairs of lines to idoxifene, tamoxifen, 3-hydroxytamoxifen, 4-iodotamoxifen and the potent imidazole-based calmodulin inhibitor, calmidazolium, showed that, whereas both of the acquired resistant cell lines exhibited around 6-fold resistance to cisplatin, no cross-resistance (resistance factor <1) was observed to all of the tamoxifen analogues and calmidazolium.

In view of previously reported synergistic effects between cisplatin and tamoxifen (McClay *et al.*, 1992, 1993*a*,*b*) we have investigated the ability of idoxifene to potentiate the cytotoxicity of cisplatin in these two paired cell lines by two independent methods. Firstly, cells were exposed concomitantly (continuous exposure) to cisplatin at various concentrations and idoxifene at a fixed, highest non-toxic concentration (2 μM) of idoxifene. Table II shows that, under these conditions, idoxifene increased the cytotoxicity of cisplatin by around 20% in the CH1 and CH1cisR6 cell lines (normalised for any growth-inhibitory effect of idoxifene alone) but decreased the cytotoxicity of cisplatin in the 41M pair of lines; resistance factors to cisplatin remained similar in the presence or absence of idoxifene. None of these differences reached statistical significance. Secondly, the effects of combining cisplatin and idoxifene were studied by median effect analysis, a statistical method whereby the interaction between two cytotoxic drugs may be evaluated using constant molar ratios (based on relative $IC_{50}$ values of the two drugs for each cell line) according to published methods (Chou & Talalay, 1984). The results are shown in Table III. Contrasting effects were observed in the two pairs of lines. While the drug combination was antagonistic (combination index $> 1$) in the 41M and 41McisR6 cell lines, weak synergistic effects were observed with CH1. A previous study (Perez et al., 1992b) used sham combinations of cisplatin and carboplatin; CI values at $Fa = 0.5$ ranged from $0.953 \pm 0.289$ to $0.935 \pm 0.271$. Therefore, the combination in the CH1cisR6 line is additive.

We have begun to investigate the possible mechanistic basis of the contrasting median effect analysis data for the two pairs of cell lines. Figure 4 shows platinum accumulation data for 41McisR6 (Figure 4a) and CH1cisR6 (Figure 4b) following a 2 h exposure of cells to differing concentrations of cisplatin either alone or in the presence of 2 μM idoxifene. Although reduced platinum uptake has previously been shown to be a major contributing factor to the acquired resistance observed in 41McisR6 cells (Loh et al., 1992) and marked antagonism (Table III) was observed with the 41McisR6 cells, there was no significant difference in

**Figure 3** Western blot of P-glycoprotein in membrane samples of (1) LX4 (positive control), (2) 41M, (3) 41McisR6, (4) 41MdoxR, (5) CH1, (6) CH1cisR6 and (7) CH1doxR using C219 monoclonal antibody.

platinum accumulation in the presence of idoxifene. Similarly, no difference in uptake was observed in the CH1cisR6 cell line.

Our previous findings have shown that acquired resistance to cisplatin in the CH1/CH1cisR pair of lines is mediated at the level of DNA, similar levels of total platinum being bound to DNA in the two cell lines following equimolar concentrations of cisplatin (Kelland et al., 1992a). Figure 5 shows the rates of formation and removal of platinum–DNA interstrand cross-links (ISCs) as measured by alkaline filter elution in the CH1 and CH1cisR6 cell lines following a 2 h exposure to cisplatin (25 μM) and allowing removal in the presence (2 μM) or absence of idoxifene. The results show that, while similar levels of ISC are formed in the two cell lines immediately after the 2 h drug exposure (time zero), at 5 h post exposure more ISCs are present in the parent CH1 cells. Removal of cross-links was greater in the acquired resistant CH1cisR6 line (90% and 15% were removed at 2 h versus 5 h in CH1cisR6 and CH1 respectively). Idoxifene, however, did not markedly affect the rate of ISC removal in either cell line.

### Discussion

Through the use of additional analogues of tamoxifen and the potent imidazole-based calmodulin inhibitor calmidazolium, we have shown a strong positive correlation between inhibition of calmodulin and *in vitro* cytotoxicity to four oestrogen receptor-negative ($<10$ fmol $mg^{-1}$ cytosolic protein) human ovarian carcinoma cell lines. These results suggest that inhibition of calmodulin, an intracellular calcium-binding protein that is known to play a key role in regulating cell proliferation (Means, 1988), may be of importance in mediating the cytotoxic effects of idoxifene. As calmodulin has been suggested as a possible target for new chemotherapeutic strategies (Hait, 1987), further variation of the alkylaminoethoxy side chain of tamoxifen to obtain even greater inhibition of calmodulin is in progress. Previous studies with the same series of compounds have shown a similar correlation between calmodulin inhibition and cytotoxicity for oestrogen receptor-positive MCF-7 human breast cancer cells but no such correlation for murine L1210 or rat Walker cells (Rowlands et al., 1990).

Tamoxifen has proven to be one of a number of diverse agents capable of reversing P-170 glycoprotein-mediated multidrug resistance (Ramu et al., 1984; Beck, 1991). One of the most studied agents in this context in the clinic has been the calcium channel blocker verapamil (Beck, 1991). Our

**Table I** Comparative effect of verapamil, tamoxifen and idoxifene on doxorubicin cytotoxicity in CH1/CH1doxR and 41M/41MdoxR cells

| | *96 h $IC_{50}$ (nM)* | | | *96 h $IC_{50}$ (nM)* | | |
|---|---|---|---|---|---|---|
| | *41M* | *41MdoxR* | *Rf*[a] | *CH1* | *CH1doxR* | *Rf*[a] |
| Doxorubicin | 39 ± 21 | 280 ± 94 | 7.2 | 5.3 ± 2.2 | 460 ± 80 | 87 |
| Doxorubicin + 6 μM verapamil | 22 ± 18 | 71 ± 40 | 3.2 | 6.6 ± 3 | 49 ± 10 | 7.4 |
| Doxorubicin + 4 μM tamoxifen | 45 ± 22 | 110 ± 40 | 2.4 | 6.9 ± 3 | 97 ± 20 | 14 |
| Doxorubicin + 2 μM idoxifene | 45 ± 20 | 85 ± 30 | 1.9 | 6.6 ± 3 | 72 ± 10 | 10.9 |

Values are mean ± s.d. in three experiments. [a]Rf, resistance factor ($IC_{50}$ resistant line/$IC_{50}$ parent line).

**Table II** Effect of idoxifene (IDOX) on cisplatin cytotoxicity in 41M/41McisR6 and CH1/CH1cisR6 cells

| | *96 h $IC_{50}$ (μM)* | | | *96 h $IC_{50}$ (μM)* | | |
|---|---|---|---|---|---|---|
| | *41M* | *41McisR6* | *Rf*[a] | *CH1* | *CH1cisR6* | *Rf*[a] |
| Cisplatin | 0.21 ± 0.02 | 1.35 ± 0.5 | 6.6 | 0.12 ± 0.02 | 0.71 ± 0.13 | 6.0 |
| Cisplatin + 2 μM IDOX | 0.31 ± 0.04 | 1.63 ± 0.25 | 5.3 | 0.09 ± 0.03 | 0.57 ± 0.15 | 6.6 |

Values are mean ± s.d. in three experiments. [a]Rf, resistance factor ($IC_{50}$ resistant line/$IC_{50}$ parent line)

**Table III** Median effect analysis of the interaction between idoxifene (IDOX) and cisplatin (CDDP) in 41M/41McisR6 and CH1/CH1cisR6 cells

| *Cell line* | *Molar ratio (CDDP–IDOX)* | *Combination index (Fa = 0.5)* | *Interaction* |
|---|---|---|---|
| 41M | 1:2 | 1.5 ± 0.6 | Antagonistic |
| 41McisR6 | 1:4 | 2.1 ± 1.1 | Antagonistic |
| CH1 | 1:4 | 0.76 ± 0.3 | Additive/synergistic |
| CH1cisR6 | 1:6 | 0.95 ± 0.4 | Additive |

Values are mean ± s.d. in three experiments.

**Figure 4** Intracellular accumulation of cisplatin in (**a**) 41McisR6 and (**b**) CH1cisR6 cells in the absence (open symbols) and presence (closed symbols) of 2 μM idoxifene.

**Figure 5** DNA interstrand cross-link repair as measured by alkaline elution immediately after 2 h exposure to cisplatin (25 μM) in the absence (open symbols) and presence (closed symbols) of 2 μM idoxifene in CH1 (○) and CH1cisR6 (△) cells.

comparative studies using two newly established acquired doxorubicin-resistant human ovarian carcinoma cell lines (shown to overexpress P-170 membrane glycoprotein) showed that idoxifene (at a concentration of 2 μM) is at least as effective as verapamil or tamoxifen in reversing resistance. At present, it is premature to make conclusions concerning the achievable plasma and tumour levels of idoxifene in patients. However, based on these preclinical data, idoxifene may also confer useful clinical benefit in the multidrug resistance setting.

We have also investigated whether idoxifene might possess a therapeutic role in modulating the effects of cisplatin. We have demonstrated non-cross-resistance to idoxifene (and other tamoxifen analogues) in two acquired resistant lines with contrasting mechanisms of resistance to cisplatin. In common with the data of McClay and co-workers with tamoxifen, weak synergism between cisplatin and idoxifene was apparent for the parent CH1 cell line. However, this synergism was cell line dependent, with only additive effects being observed for the CH1cisR6 line and antagonistic effects being observed for the 41M and 41McisR6 lines. Interestingly, McClay *et al.*, 1993*a*) observed a degree of antagonism in another melanoma cell line (Brown cell line) and in a T-289 subline selected for resistance to tamoxifen.

Thus far, synergism between tamoxifen and cisplatin has been demonstrated *in vitro* in three types of human tumour cell line: malignant melanoma (T-289 cells, McClay *et al.*, 1992, 1993*a*), ovarian (2008 cells, McClay *et al.*, 1993*b*; primary ovarian tumour, Scambia *et al.*, 1992) and small-cell lung (UMC5 cells, McClay *et al.*, 1993*b*). In common with the above findings, our studies show that synergy is not dependent on the presence of oestrogen receptors and, as observed for a cisplatin-resistant variant of the T-289 melanoma line, the synergistic interaction is less for the acquired cisplatin-resistant CH1 line than for its parent. Our studies also demonstrate that the synergy observed (and the antagonism observed for the 41M pair of lines) is not associated with two of the known main mechanisms of resistance to cisplatin (reduced drug transport and enhanced removal of platinum-induced DNA interstrand cross-links). This is in agreement with the melanoma cell line studies (McClay *et al.*, 1992, 1993*a*).

In addition to effects on oestrogen receptors, tamoxifen has also been reported to exert other effects, notably on calmodulin (Lam, 1984) and protein kinase C (O'Brian *et al.*, 1985). Moreover, calmodulin inhibitors, W-7 [(*N*-6-aminohexyl)-5-chloro-1-naphthalenesulphonamide], and the clinically used antipsychotic agent, trifluoperazine, have both been shown to potentiate the cytotoxic effects of cisplatin *in vitro* (Kikuchi *et al.*, 1990; Perez *et al.*, 1992*a*). In the trifluoperazine plus cisplatin study, synergy was observed in four of six cell lines by median effect analysis, while clear antagonism was apparent in the remaining cell lines (Perez *et al.*, 1992*a*). Intriguingly, with the W-7 study, potentiation appeared to be mediated through enhanced platinum uptake, while with trifluoperazine a mechanism involving inhibition of the incision step of DNA repair was proposed. In our studies, however, the disparate results obtained by median effect analysis did not appear to be related to either platinum uptake or repair of platinum–DNA adducts (although it should be noted that the DNA interstrand cross-links measured by alkaline elution only represent about 2% of the total platinum–DNA adducts induced by cisplatin). Furthermore, the synergy observed in the T-289 melanoma cell line did not appear to be related to effects on calmodulin (or protein kinase C) (McClay *et al.*, 1993*a*). Nevertheless, as idoxifene is a more effective inhibitor of calmodulin than tamoxifen (Rowlands *et al.*, 1990), it remains conceivable that the synergistic/antagonistic effects observed with cisplatin in this study may be related to differential effects on calmodulin. Alternatively, or in addition, effects mediated by tamoxifen on so-called type II oestrogen binding sites may be involved (Scambia *et al.*, 1992).

In summary, idoxifene showed improved cytotoxicity compared with tamoxifen in oestrogen receptor-negative human ovarian carcinoma cell lines. Based on data using acquired doxorubicin- or cisplatin-resistant cell lines and combination studies with cisplatin, idoxifene may confer useful clinical benefit in combination therapy with doxorubicin and, for some tumours, with cisplatin. However, further mechanistic studies are necessary to elucidate the underlying basis of the cell line-specific synergy or antagonism observed between idoxifene and cisplatin.

This work is supported by grants to the Institute of Cancer Research from the Cancer Research Campaign (UK) and the Medical Research Council.

### References

BECK, W.T. (1991). Modulators of P-glycoprotein-associated multidrug resistance. In *Molecular and Clinical Advances in Anticancer Drug Resistance*, Ozols, R.F. (ed.) pp. 151-170. Kluwer Academic Publishers: Boston.

BREAST CANCER TRIALS COMMITTEE & EDINBURGH SCTO (1987). Adjuvant tamoxifen in the management of operable breast cancer: The Scottish trial. *Lancet*, **ii**, 171-175.

BRUNING, P.F. (1992). Droloxifene, a new antioestrogen in postmenopausal advanced breast cancer: preliminary results of a double-blind dose finding phase II trial. *Eur. J. Cancer*, **28A**, 1404-1407.

CHANDER, S.K., MCCAGUE, R., LUQMANI, Y., NEWTON, C., DOWSETT, M., JARMAN, M. & COOMBES, R.C. (1991). Pyrrolidino-4-iodotamoxifen and 4-Iodotamoxifen, new analogues of the antioestrogen tamoxifen for the treatment of breast cancer. *Cancer Res.*, **51**, 5851-5858.

CHOU, T.C. & TALALAY, P. (1984). Quantitative analysis of dose-effect relationships: the combined effects of multiple drugs or enzyme inhibitors. *Adv. Enz. Regul.*, **22**, 27-55.

COLE, S.P.C., BHARDWAJ, G., GERLACH, J.H., MACKIE, J.E., GRANT, C.E., ALMQUIST, K.C., STEWART, A.J., KURZ, E.U., DUNCAN, A.M.V. & DEELEY, R.G. (1992). Overexpression of a transporter gene in a multidrug-resistant human lung cancer cell line. *Science*, **258**, 1650-1654.

FOSTER, A.B., JARMAN, M., LEUNG, O.T., MCCAGUE, R., LECLERCQ, G. & DEVLEESCHOUWER, N. (1985). Hydroxy derivatives of tamoxifen. *J. Med. Chem.*, **28**, 1491-1497.

FUTSCHER, B.W. & DALTON, W.S. (1993). P-glycoprotein mediated multidrug resistance. In *Drug Resistance in Oncology*, Teicher, B.A. (ed.) pp. 461-478. Marcel Dekker: New York.

HAIT, W.N. (1987). Targeting calmodulin for the development of novel cancer chemotherapeutic agents. *Anti-Cancer Drug Design*, **2**, 139-149.

HILLS, C.A., KELLAND, K.R., ABEL, G., SIRACKY, J., WILSON, A.P. & HARRAP, K.R. (1989). Biological properties of ten human ovarian carcinoma cell lines: calibration *in vitro* against four platinum complexes. *Br. J. Cancer*, **59**, 527-534.

KARTNER, N., RIORDAN, J.R. & LING, V. (1983). Cell surface P-glycoprotein associated with multidrug resistance in mammalian cell lines. *Science*, **221**, 1285-1288.

KELLAND, L.R., MISTRY, P., ABEL, G., LOH, S.Y., O'NEILL, C.F., MURRER, B.A. & HARRAP, K.R. (1992*a*). Mechanism-related circumvention of acquired *cis*-Diamminedichloroplatinum (II) resistance using two pairs of human ovarian carcinoma cell lines by ammine/amine platinum (IV) dicarboxylates. *Cancer Res.*, **52**, 3857-3864.

KELLAND, L.R., MURRER, B.A., ABEL, G., GIANDOMENICO, C.M., MISTRY, P. & HARRAP, K.R. (1992*b*). Ammine/amine platinum (IV) dicarboxylates: a novel class of platinum complex exhibiting selective cytotoxicity to intrinsically cisplatin-resistant human ovarian cell lines. *Cancer Res.*, **52**, 822-828.

KIKUCHI, Y., IWANO, I., MIYAUCHI, M., SASA, H., NAGATA, I. & KUKI, E. (1990). Restorative effects of calmodulin antagonists on reduced cisplatin uptake by cisplatin-resistant human ovarian cancer cells. *Gynecol. Oncol.*, **39**, 199-203.

LAM, P.H.-Y. (1984). Tamoxifen is a calmodulin antagonist in the activation of cAMP phosphodiesterase. *Biochem. Biophys. Res. Commun.*, **118**, 27-32.

LOH, S.Y., MISTRY, P., KELLAND, L.R., ABEL, G. & HARRAP, K.R. (1992). Reduced drug accumulation as a major mechanism of acquired resistance to cisplatin in a human ovarian carcinoma cell line: circumvention studies using novel platinum (II) and (IV) ammine/amine complexes. *Br. J. Cancer*, **66**, 1109-1115.

MCCAGUE, R., LECLERCQ, G., LEGROS, N., GOODMAN, J., BLACKBURN, G.M., JARMAN, M. & FOSTER, A.B. (1989). Derivatives of tamoxifen dependence of antioestrogenicity on the 4-substituent. *J. Med. Chem.*, **32**, 2527-2533.

MCCLAY, E.F., ALBRIGHT, K.D., JONES, J.A., EASTMAN, A., CHRISTEN, R. & HOWELL, S.B. (1992). Modulation of cisplatin resistance in human malignant melanoma cells. *Cancer Res.*, **52**, 6790-6796.

MCCLAY, E.F., ALBRIGHT, K.D., JONES, J.A., CHRISTEN, R.D. & HOWELL, S.B. (1993*a*). Tamoxifen modulation of cisplatin sensitivity in human malignant melanoma cells. *Cancer Res.*, **53**, 1571-1576.

MCCLAY, E.F., ALBRIGHT, K.D., JONES, J.A., CHRISTEN, R.D. & HOWELL, S.B. (1993*b*). Tamoxifen modulation of cisplatin cytotoxicity in human malignancies. *Int. J. Cancer*, **55**, 1018-1022.

MCKEAGE, M.J. & KELLAND, L.R. (1993). New platinum drugs. In *Molecular Aspects of Anticancer drug-DNA Interactions*, Neidle, S. & Warings, M.J. (eds) pp. 169-212. Macmillan Press: London

MEANS, A.R. (1988). Molecular mechanisms of action of calmodulin. *Recent Progr. Hormone Res.*, **44**, 223-262.

O'BRIAN, C.A., LISKAMP, R.M., SOLOMON, D.H. & WEINSTEIN, I.B. (1985). Inhibition of protein kinase C by tamoxifen. *Cancer Res.*, **45**, 2464-2465.

PEREZ, R.P., HANDEL, L.M. & HAMILTON, T.C. (1992*a*). Potentiation of cisplatin cytotoxicity in human ovarian carcinoma cell lines by trifluoperazine, a calmodulin inhibitor. *Gynecol. Oncol.*, **46**, 82-87.

PEREZ, R.P., PEREZ, K.M., HANDEL, L.M. & HAMILTON, T.C. (1992*b*). *In vitro* interactions between platinum analogues in human ovarian-carcinoma cell lines. *Cancer Chemother. Pharmacol.*, **29**, 430-434.

RAMU, A., GLAUBIGER, D. & FUKS, Z. (1984). Reversal of acquired resistance to doxorubicin in P388 murine leukaemia cells by tamoxifen and other triparanol analogues. *Cancer Res.*, **44**, 4392-4395.

ROBERTS, J.J. & FRIEDLOS, F. (1987). Quantitative estimation of cisplatin-induced DNA interstrand cross-links and their repair in mammalian cells: relationship to toxicity. *Pharmacol. Ther.*, **34**, 215-246.

ROWLANDS, M.G., PARR, I.B., MCCAGUE, R., JARMAN, M. & GODDARD, P.M. (1990). Variation of the inhibition of calmodulin dependent cyclic AMP phosphodiesterase amongst analogues of tamoxifen: correlations with cytotoxicity. *Biochem. Pharmacol.*, **40**, 283-289.

QUIGLEY, M., HAYNES, B., DOWSETT, M., JARMAN, M., HANHAM, I.W. & COOMBES, C. (1994). Phase I clinical trial of a new anti oestrogen idoxifene in advanced breast cancer. *Br. J. Cancer*, **69**, 1187.

SCAMBIA, G., RANELLETTI, F.O., PANICI, P.B., PIANTELLI, M., VINCENZO, E.D., BONANNO, G., FERRANDINA, G., ISOLA, G. & MANCUSO, S. (1992). Synergistic antiproliferative activity of tamoxifen and cisplatin on primary ovarian tumours. *Eur. J. Cancer*, **28A**, 1885-1889.

TOWBIN, H., STAEHELIN, T. & GORDON, J. (1979). Electrophoresis transfer of proteins from polyacrylamide gels to nitrocellulose sheets: procedures and some applications. *Proc. Natl Acad. Sci. USA*, **76**, 4350-4354.

TWENTYMAN, P.R., FOX, N.E., WRIGHT, K.A. & BLEEHEN, N.M. (1986). Derivation and preliminary characterisation of adriamycin resistant lines of human lung cancer cells. *Br. J. Cancer*, **53**, 529-537.